# Enteral Infant Formulas: A Narrative Review of Historical Evolution, Nutritional Composition, and Clinical Use in Pediatrics

**DOI:** 10.7759/cureus.82692

**Published:** 2025-04-21

**Authors:** Jorge Martínez-Vázquez, Ernesto Martínez-Vargas, Jeaustin Mora-Jiménez, Sebastián Arguedas-Chacón, Jonathan García-Montero, Noelia Chaves-Romero, Josué Murillo-Cubero, Esteban Zavaleta-Monestel

**Affiliations:** 1 Pediatrics, Hospital Clínica Bíblica, San José, CRI; 2 Department of Research, Hospital Clínica Bíblica, San José, CRI; 3 Faculty of Pharmacy, Universidad de Iberoamérica, San José, CRI; 4 Department of Pharmacology, University of Costa Rica, San José, CRI; 5 Health Research Unit, Hospital Clínica Bíblica, San José, CRI

**Keywords:** breastfeeding, enteral feeding, infant feeding, infant nutrition, tube feeding

## Abstract

This narrative review aims to describe the evolution, classification, nutritional composition, and clinical applications of enteral infant formulas, with an emphasis on pediatric patients who are unable to be breastfed or follow conventional feeding methods. The primary objective is to analyze the different types of formulas available - polymeric, hydrolyzed, elemental, and blenderized - and their indications based on clinical scenarios and specific patient needs.

The methodology used was a non-systematic narrative review. Relevant scientific literature was selected through a targeted search of databases such as PubMed, Scopus, ScienceDirect, and Google Scholar, including publications from the last 20 years in both English and Spanish. Key search terms included “enteral nutrition,” “infant formula,” “nutritional therapy,” and “pediatric enteral feeding.”

The article traces the historical development of infant nutrition, from breastfeeding and wet nursing to modern enteral formulas. It explains the macronutrient composition of each formula type, their preparation methods, palatability, osmolality, and caloric density. Clinical considerations and recommended formulas for specific diseases - such as obesity, cow’s milk allergy, Crohn’s disease, eosinophilic esophagitis, necrotizing enterocolitis, short bowel syndrome, and cystic fibrosis - are discussed in detail.

Additionally, the review outlines the regulatory context in the United States and Europe, noting that these products are classified as medical foods and not pharmaceuticals, and are thus subject to distinct safety, composition, and labeling requirements.

The review concludes that although breastfeeding remains the gold standard in infant nutrition, enteral formulas are indispensable in many clinical situations. Future research is expected to focus on the development of more targeted, functional, and personalized formulas to address the needs of vulnerable pediatric populations.

## Introduction and background

The history of pediatric nutrition is marked by a continuous evolution. Infant nutrition practices began with breastfeeding, but have expanded to include innovations such as the modern bottle and the use of alternatives to breast milk and formulas [1]. The invention of modern infant formulas arose from the need to address infant feeding challenges such as breastfeeding problems and the lack of nutritional alternatives to breast milk. Early attempts to create “baby foods” were nutritionally inadequate, as infants were fed primarily carbohydrate-rich diets consisting of mixtures of cereals, flour, and bread cooked in broth, water, or animal milk when breast milk was not available [2].

Over time, infant formulas evolved as scientists analyzed the composition of human breast milk, allowing animal milk, primarily cow’s milk, to be modified to more closely resemble human milk [3]. In the United States, organizations such as the American Society for Parenteral and Enteral Nutrition (ASPEN) promote and recommend the safe and appropriate use of infant formulas, taking into account the dietary needs of infants and chronic medical conditions [4]. Globally, the World Health Organization (WHO) provides guidance to governments to prevent malnutrition among infants and young children [5].

In Latin America, WHO recommendations are not always followed, increasing the risk of undernutrition and child morbidity due to inadequate breastfeeding practices and inadequate introduction of complementary feeding [6]. Historically, the region has struggled with high rates of child undernutrition, as evidenced in the 1970s when records show that one-third of children under five years of age died from undernutrition [3]. Malnutrition is associated with 2.7 million infant deaths per year, accounting for about 45% of all infant deaths [7]. Aggressive marketing of infant formula and breastmilk substitutes, combined with social and economic changes, has had a negative impact on breastfeeding rates and has led to increased commercialization of infant formula [8]. Social inequality also plays a key role in this trend, with studies indicating that formula use is higher in lower-income groups. Furthermore, in developing countries, social networks and influencers have been associated with inappropriate feeding practices, with women often relying on informal sources of information on pediatric nutrition [9].

WHO and the United Nations International Children’s Emergency Fund (UNICEF) recommend exclusive breastfeeding (EBF) for the first 6 months of life to ensure optimal development and growth [4]. For preterm infants, the preferred method of nutrition is the administration of breast milk through nasogastric tubes, as breast milk not only provides nutrition but also protection against infection and supports neurological development [10]. Premature births disrupt the development of the newborn’s organs, including the intestines, and enteral feeding helps promote the growth and maturation of the intestinal tract [11]. Complementary feeding is recommended after six months. At this point, the caloric and nutritional needs of a growing infant exceed those provided by breast milk alone [11]. In cases where breastfeeding is not possible, appropriate infant formulas are recommended.

Given the critical role of enteral nutrition in pediatric health, a comprehensive understanding of its historical development, compositional nuances, and clinical applications is essential for informed decision-making in healthcare. This narrative review aims to examine the historical evolution of infant feeding practices, analyze the compositional differences among enteral formulas, and explore their clinical indications and considerations in pediatric care.

## Review

Methodology

The bibliographic search was carried out independently by two authors in the PubMed, Scopus, and Google Scholar databases, covering the period from January 2000 to March 2025. The following keywords were used alone or in combination: infant formula, enteral nutrition, breastfeeding, pediatric enteral feeding, human milk substitutes, clinical considerations, nutritional composition, protein hydrolysate formulas, amino acid-based formulas, blenderized formulas, infant malnutrition, and disease-specific nutrition. Narrative and systematic review articles, randomized clinical trials (RCTs), clinical guidelines, observational studies, and consensus documents relevant to the clinical use and composition of enteral formulas in the pediatric population were included. In addition, the bibliographies of the selected articles and the references included in previous reviews were reviewed in order to identify additional relevant literature. Finally, the articles were selected by revision cross between both authors, prioritizing the studies with greater methodological rigor and clinical applicability. This strategy allowed us to integrate the most relevant and updated evidence on the historical evolution, classification, nutritional characteristics, and clinical applications of infant enteral formulas.

Although a formal assessment of the quality of evidence (e.g., GRADE) was not performed due to the narrative nature of this review, efforts were made to prioritize high-quality sources. Preference was given to systematic reviews, clinical guidelines, RCTs, and articles published in peer-reviewed journals indexed in major databases. This approach aimed to ensure the inclusion of reliable and clinically relevant information, even in the absence of a structured evidence-grading system.

History of pediatric nutrition

Breastfeeding and Wet Nurses

Breastfeeding was regarded as the best feeding option for newborns and infants. Extensive research and evidence over the years have confirmed the importance of breast milk in the development and growth of infants, as breast milk can modify the function of the immune system, gastrointestinal tract, and brain development [12]. However, there are exceptional cases in which the mother is unable to breastfeed her child, such as lactation failure related to physiological reasons or death of the mother during or after childbirth. Before the 20th century, it was understood that an infant who was not breastfed would eventually die, and that breast milk was the only option for sustaining a developing baby [3].

Wet nursing is considered the first alternative feeding method for infants who could not be breastfed by their own mother, and the practice quickly became key to the human societies of the time. At times, it was believed that the wet nurse could cure or sicken the baby with her breast milk. Historical documents provide information on the dietary practices of wet nurses, describing the specific foods they were encouraged to consume or avoid based on the health of the baby. In addition to dietary restrictions, wet nurses often underwent medical treatments, such as bloodletting or purging, as if they were the ones suffering from the baby’s illness [1].

First Alternatives to Breast Milk

As stated earlier, experts correctly assessed that for infants to have a healthy development, the need to find an alternative similar to human milk became a necessity. Naturally, these artificial feeding options were multiple types of animal milks, with cow’s milk being the most common. These types of milks were fed to the infant using a variety of devices and containers made of various materials, such as cow horns, clay, pottery, and wood [1]. The absence of reliable alternatives to breast milk contributed significantly to infant mortality. This is exemplified by the high mortality rate in London between 1780 and 1816, where seven out of eight hand-fed infants aged two years or less did not survive [3]. A more recent example is provided by a study in Mexico, which estimated that inadequate breastfeeding practices are associated with between 933 and 5,796 infant deaths annually due to illnesses such as respiratory infections, otitis media, gastroenteritis, necrotizing enterocolitis (NEC), and sudden infant death syndrome (SIDS). These data reinforce the ongoing importance of breastfeeding and highlight how its absence continues to impact infant mortality, even in contemporary settings [13].

Animal milk is not like breast milk in terms of macronutrient composition, and this led to the creation of homemade food supplements to use in conjunction with animal milk. In Europe, mothers and caregivers fed infants and children with substances such as panada and pap [1]. The invention of the glass bottle in the mid-19th century played an important role in improving hygiene and infant nutrition. This innovation, combined with the availability of infant formula, made bottle feeding a more attractive alternative to wet nursing for many mothers and caregivers [3].

Early Infant Formulas

The focus of early research in the late 19th and 20th centuries on infant nutrition was to create an alternative that was similar to breast milk on a nutritional level. As such, scientists began researching ways to reformulate animal milk to mimic human milk as closely as possible. The first patented and sold infant formula was developed in 1864 by Justus von Liebig and used modified cow’s milk as a base [1]. Known as “baby soup,” this formula consisted of a mixture of cow’s milk, malted starch, and potassium bicarbonate [3]. Liebig’s formula was a great success and was the inspiration for many other competitors, however, a lack of understanding of the composition of human milk meant that many of these new formulas failed. In the following decades, there was significant development and marketing of various general milk formulas and specialized formulas with different amounts of nutrients and components [2].

Early Enteral Nutrition and Feeding

Enteral nutrition (EN) consists of the administration of liquid nutritional solutions directly into the gastrointestinal tract by means of probes. Although its historical origins date back to ancient civilizations, it was not until the 16th century that its use with rudimentary instruments was formally documented [14]. However, it was not until the 1950s that permanent nasogastric tubes were implemented, despite initial resistance from the medical community, which contributed to episodes of hypoglycemia and malnutrition in infants [10]. In the 1960s, enteral formulas designed specifically for pediatric use began to be manufactured, marking a milestone in the nutritional management of the critically ill neonate [15]. Current evidence supports that early EN in preterm infants improves intestinal maturation, favors the establishment of the microbiome, and stimulates neurodevelopment, consolidating it as a key therapeutic strategy [11].

Infant Formulas

**Figure 1:**
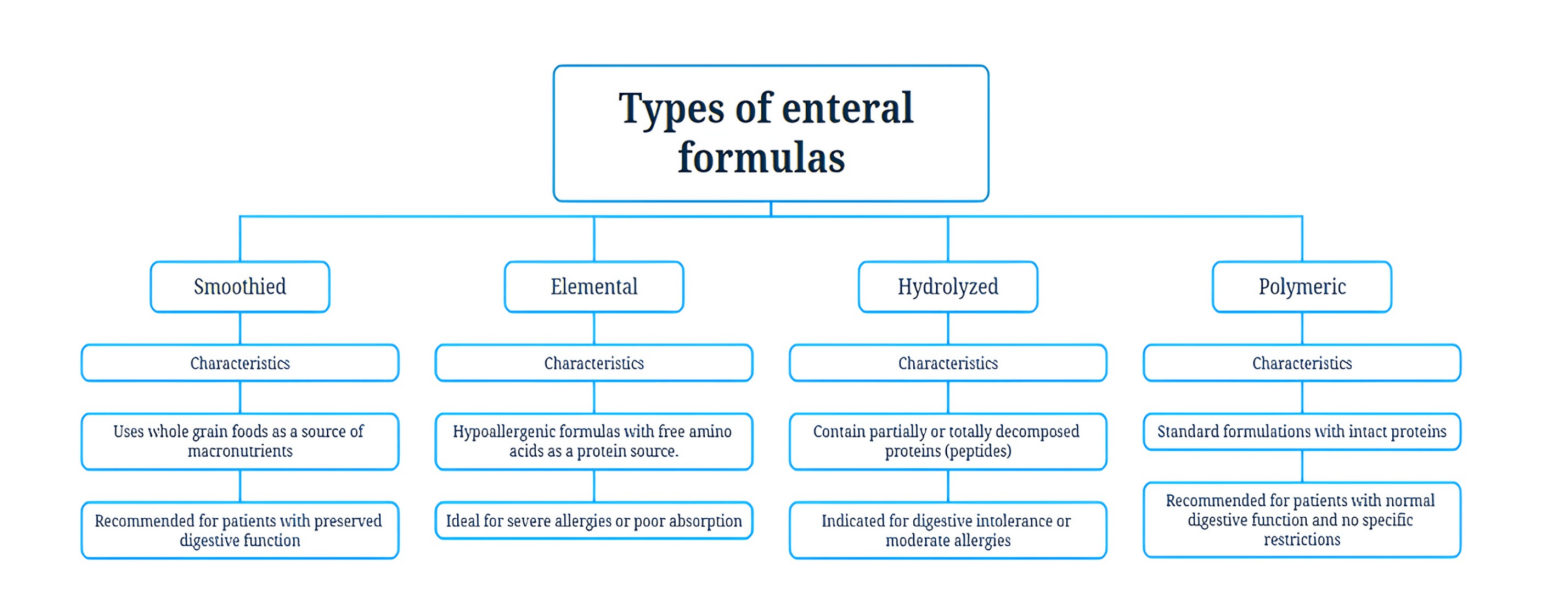
Types of enteral formulas available worldwide, characteristics, and indications. Source: Prepared by the authors.

The design of infant formulas has evolved toward highly specialized compositions that include precise mixtures of milk proteins, carbohydrates such as lactose or other sugars, lipids of vegetable origin, micronutrients, and specific additives [16]. These products are adjusted to the metabolic and clinical needs of healthy infants and those with chronic pathologies [17]. The ability to modify the concentration of macro- and micronutrients has allowed the development of personalized formulas that optimize digestive tolerance and reduce complications associated with diseases such as renal failure, intestinal disorders, and congenital heart disease [3]. There are different types of enteral formulas available, as well as their respective characteristics and indications (Figure 1).

Composition

Fat

Fat is the main energetic macronutrient in infant formulas, especially critical in premature infants, due to its role in neurological development and maturation of the central nervous system. Essential fatty acids such as linoleic acid and alpha-linolenic acid, precursors of arachidonic acid (ARA) and docosahexaenoic acid (DHA), are essential for cognitive and motor development and modulation of inflammatory responses. Both ARA and DHA are associated with a lower incidence of bronchitis, allergies, and respiratory infections [16].

Current formulas also incorporate milk fat globule membranes (MFGM), which not only facilitate lipid transport but also offer immunoprotective and neurodevelopmental functions [18]. The inclusion of structured triglycerides, both medium-chain (MCT) and long-chain (LCT), improves lipid absorption, modulates gut microbiota, reduces inflammation, and promotes bone mineralization. Common sources include fish oil and vegetable oils for MCT and canola, sunflower or soybean oil for LCT [16,17].

Protein

Proteins are fundamental in infant nutrition, since their amino acids favor tissue growth, enzyme synthesis, and neurological development. In the infant formula industry, cow's milk is the most widely used raw material due to its availability, stability in the supply chain, and nutritional profile, being a source of proteins such as whey and casein, which also have immunomodulatory properties. However, these proteins have a lower quality than those of breast milk, due to a lower content of essential amino acids [19,20].

Protein quality in formulas is key to cover the metabolic requirements of the infant, and its regulation is strict, since an excess has been associated with a greater risk of infant obesity. Currently, alternative protein sources have been incorporated, such as soy or pea, which are useful in cases of intolerance, although their use should be evaluated with caution. Mixed formulas (animal and vegetable) are not suitable for infants with cow's milk protein allergy. In addition, soy-based formulas are contraindicated in infants under six months of age due to possible exposure to phytoestrogens and their potential endocrine effects [16,19].

Carbohydrates

Lactose is the main carbohydrate in breast milk and is used in formulas to replicate its natural concentration. However, many standard pediatric formulas use corn syrup derivatives, making them lactose- and gluten-free, while others use sources such as rice syrup, pea starch, or agave. Legislation, such as that of the European Union, also allows the use of maltose, glucose, and sucrose. Among the most relevant carbohydrates are human milk oligosaccharides (HMOs), absent in animal milk, with key functions such as protection against pathogens, immune development, and promotion of a healthy intestinal microbiota [15,16].

Carbohydrates are classified into glycemic, which raise plasma glucose upon absorption in the small intestine, and nonglycemic, which reach the large intestine undigested and exert prebiotic effects. Breast milk contains more than 200 HMOs with these properties, which are essential for the intestinal biome of the infant. In an attempt to bring formulas closer to the functional properties of breast milk, many are supplemented with bovine HMOs, synthetic HMOs and probiotics, which improve intestinal colonization, reduce the incidence of diarrhea and favor gastrointestinal maturation of the infant, especially in vulnerable populations such as premature infants or immunocompromised patients [12,16,20].

Micronutrients

**Figure 2:**
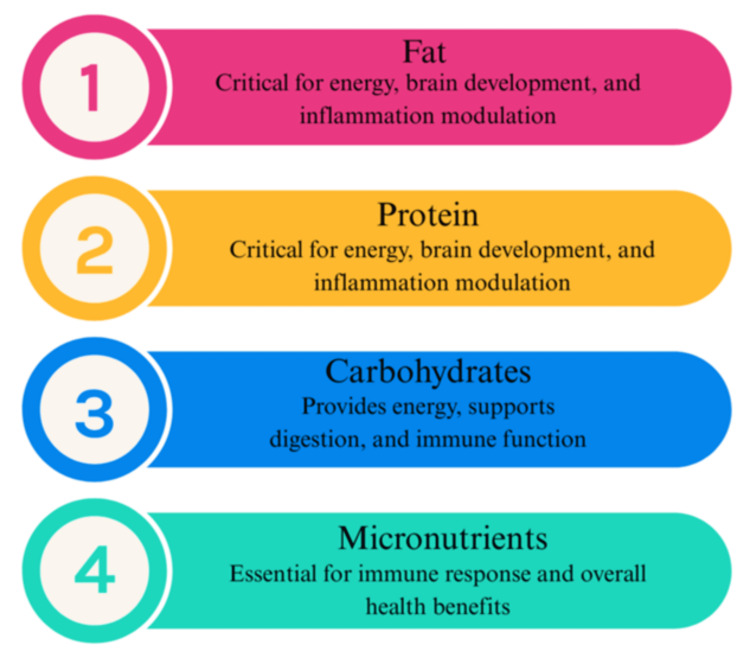
Key Components in the Nutritional Composition of Infant Formulas. Source: Prepared by the authors.

Vitamins, minerals, and trace elements are essential micronutrients that must be supplemented through diets, as the body may not be able to synthesize them. Vitamins and minerals are important supplements found in infant formulas, as both can elicit immunogenicity, a specific response that triggers the immune system to induce immune responses [16]. The Food and Drug Administration (FDA) implemented a rule that micronutrients must be present in all infant formulas, including the following vitamins: A, B1, B6, B12, C, D, E, and K along with other components such as folic acid, niacin, iron, zinc, iodine, magnesium, calcium, phosphorus, potassium, chloride, and pantothenic acid [1]. These ingredients have the necessary benefits not only to assimilate human milk, but also add healthy benefits to the formula, such as promoting the development of the immune system, brain functions, and antibody production, among others [16]. The composition of EN formulas is presented in Figure 2.

Relevant aspects for formula selection

Pediatric formulas are liquid nutritional products designed for infants and children who are unable to feed conventionally due to medical, anatomical, or functional conditions. Proper selection is essential to prevent malnutrition and should take into account both the patient’s clinical status and digestive capacity. These formulas come in three forms: powder (requires dilution, more economical), concentrated liquid (requires mixing with an equal amount of water), and ready-to-use (no preparation needed, but more expensive) [13].

According to the classification by the American Society for Parenteral and Enteral Nutrition (ASPEN), pediatric formulas are categorized as blenderized, elemental, hydrolyzed, and polymeric, depending on their degree of processing and digestive adaptability. This categorization allows nutritional support to be tailored to the specific needs of each pediatric patient [21]. Table 1 outlines the macronutrient sources according to the type of enteral formula.

**Table 1:** Specific sources of macronutrients in enteral formulas. MCT: medium-chain triglyceride; DHA: docosahexaenoic acid Source: Prepared by the authors.

Type of Enteral Formula	Source of Protein	Carbohydrate Source	Source of Fat	Main Clinical Considerations
Polymeric	Soy protein isolate, milk protein concentrates, whey concentrate	Maltodextrin, corn syrup, fructose, sucrose	MCT, canola oil, soy lecithin safflower oil, soybean oil	Standard, complete, and cost-effective formulas. Indicated for patients with normal digestive function and general nutritional needs. Good palatability
Blenderized	Whole liquefied foods (fruits, vegetables, meats, legumes, etc.)	Fruits, vegetables, cooked cereals, legumes, legumes, etc.	Natural vegetable oils (canola, olive, corn), according to homemade or commercial preparation	Natural and personalized alternative. Improves gastrointestinal symptoms and microbiome. Requires dietary supervision due to microbiological risk.
Elemental	Free amino acids (without peptide bonds)	Maltodextrin, modified corn starch, potato starch, tapioca starch	MCT Soybean oil Coconut oil Safflower oil Sunflower oil	100% hypoallergenic formulas. Indicated in severe allergies, malabsorption, short intestine, and galactosemia. High cost, low palatability.
Hydrolyzed	Hydrolyzed casein, hydrolyzed whey protein, hydrolyzed pea protein	Cornstarch maltodextrin, brown rice syrup, corn maltodextrin	Soy lecithin, sunflower lecithin, fish oil, MCT, soybean, canola, and sunflower oil	Indicated for intolerance or moderate malabsorption. Improves digestion and absorption. Some contain DHA, antioxidants, and fiber. An intermediate alternative between standard and elemental formulas.

Polymeric

Polymeric formulas are considered the standard and most cost-effective option for EN in children aged 1 to 13 years who have an intact gastrointestinal function but are unable to meet their nutritional requirements through a regular diet. These formulas are designed to serve as the sole source of complete nutrition, aligning with intake recommendations for healthy individuals. Their composition includes intact macronutrients (proteins, carbohydrates, and fats), although the specific sources of each macronutrient may vary by manufacturer. Carbohydrates are commonly derived from maltodextrin or corn syrup; proteins from caseinates or soy protein isolates; and fats from vegetable oils such as canola or soy. Additionally, these formulas include various types of soluble and insoluble fibers to support digestive health and intestinal transit, which is particularly beneficial for patients experiencing constipation or diarrhea related to EN support [15,17,21].

Blenderized Formulas

Blenderized formulas, also known as blenderized tube feeding (BTF), are composed of whole foods such as fruits, vegetables, meats, and legumes processed into a puréed or liquid form. They are perceived by caregivers as a more natural alternative, aligned with the trend toward minimally processed food consumption. Although these formulas can be commercially prepared or homemade, guidance from a registered dietitian is recommended to ensure nutritional adequacy and microbiological safety. Due to their high viscosity, adjustments are needed depending on the route of administration [15,22,23].

These formulas offer greater variability in nutrient sources and fiber content, which may promote a more diverse gut microbiome. They have been associated with reduced gastrointestinal symptoms (vomiting, diarrhea, nausea, abdominal pain) and higher caregiver and patient satisfaction. Additionally, they allow for dietary customization in cases of specific allergies or intolerances, such as lactose or soy. Some manufacturers encourage rotating blends to simulate a balanced and varied homemade diet [15,22,24,25].

Elemental Formulas

Elemental formulas, also known as amino acid-based formulas, are composed exclusively of free amino acids without peptide bonds, making them the most hypoallergenic option available for infants and children. As they contain no intact proteins or lactose, they are especially indicated for patients with galactosemia, severe lactose intolerance, multiple food allergies, or immune-mediated reactions, since their components are not recognized by T cells, thereby avoiding allergic responses [15,26].

These formulas differ from hydrolyzed formulas in that their protein source is completely broken down, and they typically have a higher proportion of MCTs, which enhances intestinal absorption. Although they are a vital therapeutic tool in cases of severe malabsorption, short bowel syndrome, or other complex digestive disorders, their use may be limited by cost, as they can be twice as expensive as polymeric formulas [15,27].

Hydrolyzed Protein Formulas

Hydrolyzed formulas, also referred to as semi-elemental or peptide-based formulas, contain partially digested proteins broken down into short-chain peptides and free amino acids, facilitating digestion and absorption. They are indicated for infants and children with malabsorption or gastrointestinal intolerance to polymeric formulas. The proteins used may be derived from whey, casein, or pea protein, allowing for selection based on the individual patient’s tolerance [18].

As for carbohydrates, these formulas often include maltodextrin, cornstarch, sugar, or combinations thereof. Additionally, many peptide-based formulas are enriched with essential antioxidant micronutrients (such as vitamins C, D, and E, selenium) and omega-3 fatty acids like DHA, which support immune and neurological development. Although some authors group hydrolyzed and elemental formulas together due to the shared hydrolysis process, in clinical practice, they are typically distinguished, as elemental formulas contain only free amino acids and no peptides [26].

Considerations for proper formula selection

Formula Preparation

Certain considerations regarding each formula form include preparation requirements. Powdered formulas are not considered sterile and are more prone to mixing errors, whereas ready-to-feed formulas are stable until opened, have a shorter shelf life, and require refrigeration [15]. The water used to reconstitute powdered or dilute liquid formulas is also important; the temperature should not exceed 40°C to prevent denaturation and destruction of proteins [19].

Consumption Considerations

In pediatric EN, formula viscosity and consistency are critical factors-especially when using small-bore feeding tubes such as nasogastric or jejunostomy tubes. Formulas that are too thick may clog the tube, whereas appropriate viscosity reduces that risk. However, thicker formulas tend to be better tolerated in patients with gastroesophageal reflux [15,21].

Palatability also influences formula selection when administered orally. Polymeric formulas are generally the most palatable and are available in a variety of flavors. In contrast, elemental formulas tend to have poor palatability due to their high content of branched-chain amino acids, which are known for their bitter taste. Some manufacturers include additives such as citric acid to improve flavor. However, when the formula is administered via direct enteral feeding (e.g., prolonged tube feeding), palatability becomes irrelevant, and some formulations are intentionally bland for such use [15,28].

Osmolality and Caloric Density

Osmolality is a key parameter in enteral formulas, as it reflects the concentration of solutes per kilogram of solvent. High osmolality can lead to adverse effects such as osmotic diarrhea-especially in infants-by drawing water into the intestinal lumen due to unabsorbed osmoles. This risk increases in hospitalized patients who receive high-osmolality medications alongside enteral feeding. Moreover, hypercaloric formulas tend to have higher osmolality due to their greater nutrient concentration [15].

On the other hand, caloric density is a crucial factor in pediatric nutrition, as it directly affects growth and development. There are low-calorie formulas designed for infants with low metabolic demands; however, these may require additional supplementation to meet essential micronutrient needs. Balancing osmolality, gastrointestinal tolerance, and energy density is fundamental in selecting the appropriate formula for each patient [21].

Disease-Specific Considerations

**Figure 3:**
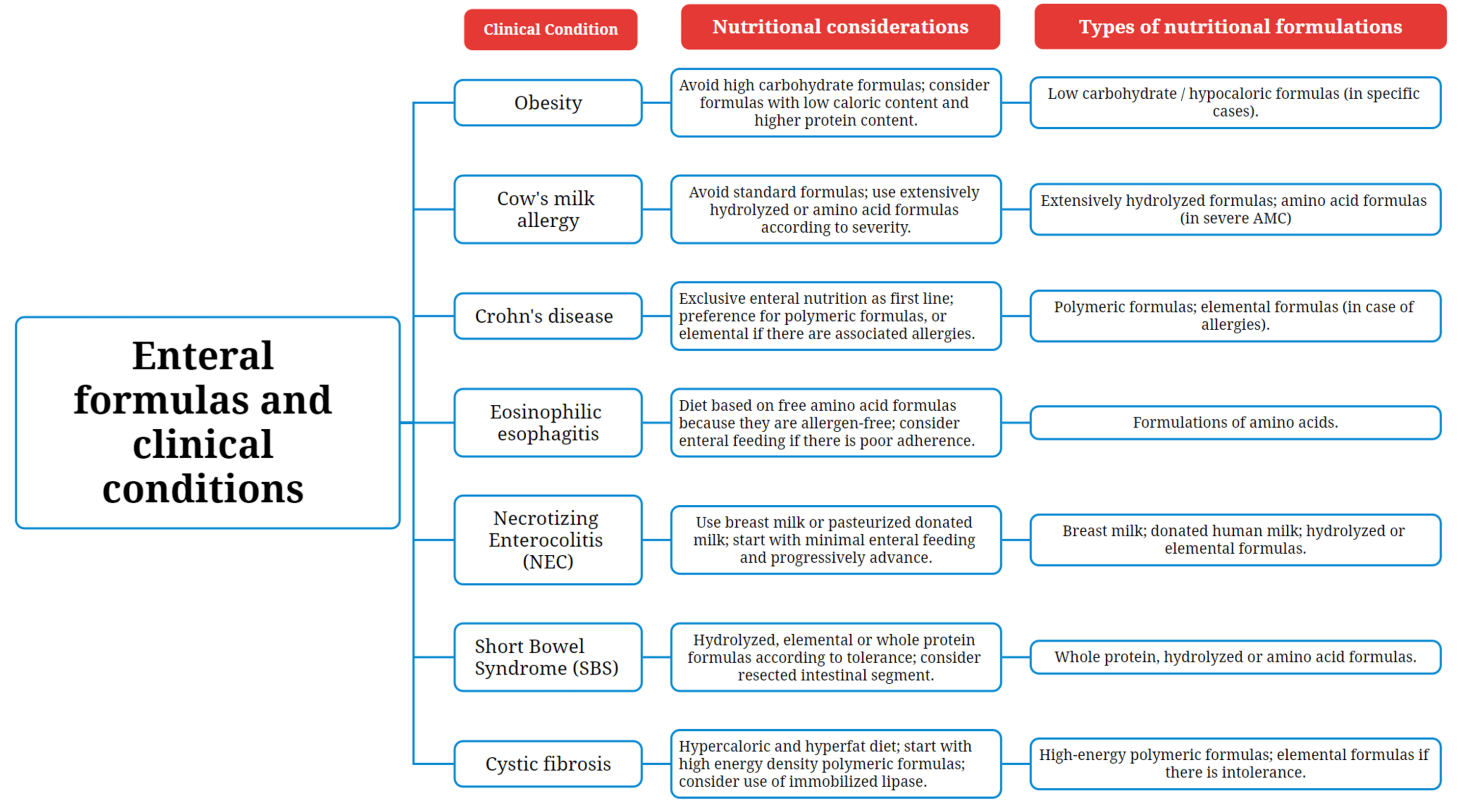
Clinical Conditions and Recommended Enteral Formulas in Pediatric Patients. Source: Prepared by the authors.

A brief summary can be seen below (Figure 3), highlighting the different diseases, nutritional considerations, and recommended formulas, which will be explored in greater detail.

Obesity

The first months of life represent a critical window for preventing future metabolic, cardiovascular, and obesity-related diseases. Growth trajectory and weight gain during the first year of life are strongly correlated with the risk of obesity later in life. Therefore, in pediatric hospital settings, systematic nutritional screening is recommended to identify patients who require a comprehensive nutritional assessment and an individualized care plan [29].

Animal studies have shown that formulas with a high carbohydrate content can lead to hyperinsulinemia and adult-onset obesity. In this regard, certain formulas developed for adults with obesity-characterized by reduced carbohydrate content and a higher protein proportion-may be considered for children with low energy requirements, always under professional supervision. In preterm infants requiring EN, breast milk should be initiated as early as possible. Furthermore, early introduction of infant formulas (before 3 months of age) has been associated with accelerated growth and a higher body mass index (BMI) in adulthood [20].

Cow’s milk allergy (CMA)

Cow’s milk protein allergy (CMA) is the most common food allergy in infants and can be classified into IgE-mediated forms (with immediate onset) and non-IgE-mediated forms (with delayed onset, often mimicking other gastrointestinal disorders). Standard infant formulas contain proteins derived from cow’s milk and should be avoided in patients with a confirmed CMA diagnosis. In some cases, soy-based formulas are used as alternatives, although their use should be medically supervised, particularly in infants under 6 months of age [30,31].

Extensively hydrolyzed formulas are the first-line treatment for infants with mild to moderate allergies, as they contain low-molecular-weight peptides with reduced allergenicity, potentially promoting the development of oral tolerance. However, their poor palatability and high cost may limit caregiver acceptance. In cases of severe CMA or multiple food allergies, amino acid-based formulas are the safest option, as they are completely free of dairy proteins. As a preventive measure, breastfeeding mothers may also be advised to eliminate cow’s milk from their own diets. Finally, although partially hydrolyzed formulas have shown some preventive potential, breastfeeding remains the optimal feeding option for all infants, including those at high allergy risk [32,33].

Crohn’s disease

Crohn’s disease (CD) is a chronic and recurrent form of inflammatory bowel disease (IBD), characterized by transmural inflammation of the gastrointestinal tract, most commonly affecting the terminal ileum and colon. In pediatric patients, clinical presentation can differ from adults, with isolated colonic inflammation being more common. The disease impairs nutrient absorption and, along with abdominal pain and reduced appetite, significantly increases the risk of malnutrition and weight loss [34-36].

In this context, exclusive enteral nutrition (EEN) is recommended as the first-line treatment for inducing remission in children, proving to be more effective and safer than corticosteroid therapy in achieving mucosal healing. Polymeric formulas are preferred due to better palatability and ease of administration, including oral intake. Elemental formulas may be used in patients with associated food allergies, such as CMA, although no significant differences in efficacy have been observed between the formula types in inducing remission. The therapeutic goals include improving nutritional status, controlling symptoms, and maintaining clinical remission of active disease [35,36].

Eosinophilic esophagitis

Eosinophilic esophagitis is a chronic inflammatory disease of the esophagus with an immunologic origin, characterized by eosinophilic infiltration that leads to esophageal dysfunction. In pediatric patients, it may present with dysphagia, odynophagia, food impaction, and feeding disorders. Given its association with non-IgE-mediated food allergies, nutritional treatment typically focuses on eliminating specific allergens from the diet [15].

Elemental diets, based on free amino acid formulas, are an effective therapeutic option as they are completely allergen-free. However, poor palatability may hinder adherence, and therefore, enteral administration is often used, especially in patients with feeding resistance or high nutritional risk [37].

Necrotizing enterocolitis (NEC)

Necrotizing enterocolitis (NEC) is a severe and potentially life-threatening gastrointestinal disease that primarily affects premature newborns due to the immaturity of their digestive, immune, respiratory, and cardiovascular systems. NEC is characterized by severe intestinal inflammation that may progress to intestinal necrosis. Given the high risk in this population, EN must be managed with extreme caution [38].

Current recommendations promote the exclusive use of the mother’s own breast milk, and when unavailable, pasteurized donor human milk. This approach has been shown to significantly reduce the risk of NEC due to the immunologic and protective components found in breast milk. Minimal EN (trophic feeding) is advised to begin within the first 48 hours of life and should be progressively increased in volume and frequency based on the infant’s clinical tolerance [39].

Once tolerance is established, feeding should be advanced over 3 to 7 days. Transitioning to full enteral feeding may involve the use of preterm infant formulas as well as hydrolyzed, semi-elemental, or elemental formulas, which offer benefits in digestion, absorption, and reduction of allergic reactions. Nevertheless, concerns remain among some clinicians about the potential increased risk of NEC with formula use in preterm infants, highlighting the need for further research and clinical trials on specialized formulas in this vulnerable population [40,41].

Short bowel syndrome (SBS)

Short bowel syndrome (SBS) is the leading cause of intestinal failure in the pediatric population and is characterized by a significant reduction in the length or function of the intestine, which prevents adequate nutrient absorption through conventional oral feeding. This condition may result from extensive surgical resections or congenital malformations of the gastrointestinal tract, leading to complications such as malnutrition, diarrhea, malabsorption, growth delay, and fluid-electrolyte imbalances [15,42].

EN is a cornerstone in the intestinal rehabilitation process and should be initiated as early as possible to promote functional adaptation of the remaining bowel. In infants, human milk is the preferred option. For older children or those with feeding intolerance, enteral formulas such as whole protein formulas, extensively hydrolyzed formulas, or amino acid-based formulas may be used, depending on the degree of malabsorption, intestinal tolerance, and clinical needs. Formula selection should take into account the length and location of the resected intestinal segment, as this directly influences the type and severity of nutritional deficiencies [15,42].

Cystic fibrosis (CF)

Cystic fibrosis (CF) is an autosomal recessive genetic disorder that affects chloride transport at the cellular level, resulting in thick mucus production that compromises both respiratory function and pancreatic and intestinal function. In the digestive context, this leads to fat malabsorption, weight loss, and malnutrition-especially in children under two years of age-where enteral feeding becomes a critical intervention [15,43].

Nutritional management should focus on a high-calorie, high-fat diet, supplemented with pancreatic enzyme replacement therapy to enhance nutrient absorption. While there is no specific formula designed exclusively for CF, high-energy polymeric formulas are generally recommended as a first option. In cases of gastrointestinal intolerance, hydrolyzed or elemental formulas may be considered. The use of immobilized lipase cartridges in patients receiving EN has been associated with reduced gastrointestinal symptoms such as abdominal distension, steatorrhea, and gastrointestinal discomfort, thus improving adherence to nutritional therapy [15,43].

International regulations on enteral formulas

EN formulas are classified as medical foods by the USFDA and are not subject to the same stringent regulatory standards as pharmaceutical products. This means they do not require premarket review or approval, as medications do. According to the FDA, a medical food is defined as a food formulated to be consumed or administered enterally under medical supervision, and intended for the specific dietary management of a disease or condition for which distinctive nutritional requirements are established by recognized scientific principles and evaluated by a healthcare professional [44].

**Figure 4:**
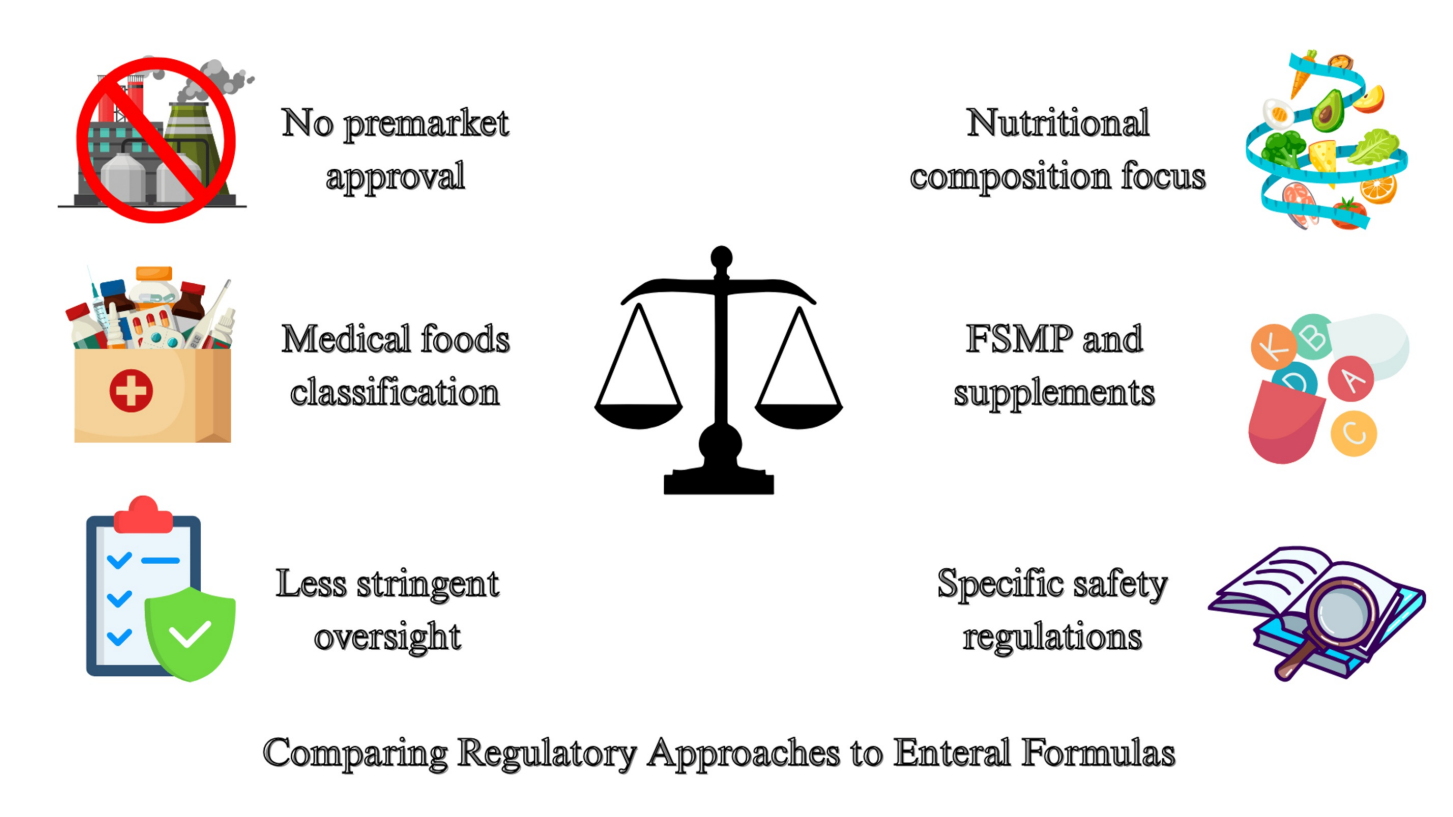
Regulatory Contrast Between U.S. and European Standards for Enteral Infant Formulas. FSMP: foods for special medical purposes Source: Prepared by the authors.

In the European context, the European Medicines Agency (EMA) classifies EN products as foods for special medical purposes (FSMP) and dietary supplements (Figure 4). As in the USA, these products are not formally registered as medicinal drugs, which impacts the level of regulatory oversight, patient access, and reimbursement policies. Nevertheless, they are subject to specific regulations that ensure their safety, nutritional composition, and labeling, guaranteeing that they meet the individual nutritional needs of their target users [45].

Regarding infant nutrition, foods intended for infants and young children are regulated by specific directives from the European Commission. These directives mandate that products be safe, possess specialized nutritional composition, be clearly distinguishable from conventional foods, and meet the specific nutritional requirements of this population. As such, the strict regulation of infant formulas used during the first months of life is crucial to ensure adequate and safe nutrition during infancy [46].

## Conclusions

Infant nutrition has undergone remarkable progress, evolving from traditional practices to modern, scientifically formulated approaches. While breastfeeding remains the ideal method of feeding for infants, infant formulas serve as a crucial alternative for those with specific medical or nutritional needs, or in contexts where breastfeeding is not feasible. Current formulations are designed to closely replicate the composition of human milk, offering safe and effective nutritional support through the integration of advanced scientific knowledge. However, the selection of an appropriate formula must be individualized, considering factors such as the infant’s age, clinical condition, digestive tolerance, osmolality, and caloric density.

Although enteral infant formulas are not regulated as pharmaceutical drugs, agencies such as the USFDA and the EMA have established frameworks to ensure product safety, quality, and appropriate labeling, especially for formulas intended for vulnerable populations. As the science of nutrition continues to evolve, there is a growing need for sustained research and innovation aimed at enhancing the functional and personalized aspects of infant formulas. Future developments should strive to address the dynamic and complex nutritional demands of infants, particularly those with chronic diseases or high-risk conditions, through increasingly specialized and evidence-based formulations.

## References

[1] Stevens EE, Patrick TE, Pickler R (2009). A history of infant feeding. J Perinat Educ.

[2] Kunz C (2012). Historical aspects of human milk oligosaccharides. Adv Nutr.

[3] (2025). History of Pediatric Nutrition and Fluid Therapy | Pediatric Research. https://www.nature.com/articles/pr2003494.

[4] Ferreira CS, Azeredo CM, Rinaldi AE (2021). Trends in social inequalities in breastfeeding and infant formulas in Latin American countries between the 1990 and 2010 decades. Public Health Nutr.

[5] (2025). Global strategy for infant and young child feeding. https://www.who.int/publications/i/item/9241562218.

[6] Ladino L, Sánchez N, Vázquez-Frias R, Koletzko B (2021). Latin American considerations for infant and young child formulae. Nutrients.

[7] (2025). Infant and young child feeding. https://www.who.int/news-room/fact-sheets/detail/infant-and-young-child-feeding.

[8] Meira CA, Buccini G, Azeredo CM, Conde WL, Rinaldi AE (2023). Infant feeding practices in three Latin American countries in three decades: what demographic, health, and economic factors are relevant?. Front Nutr.

[9] Ahern GJ, Hennessy AA, Ryan CA, Ross RP, Stanton C (2019). Advances in infant formula science. Annu Rev Food Sci Technol.

[10] Webbe J, Uthaya S, Modi N (2022). Nutrition for the micro preemie: Beyond milk. Semin Fetal Neonatal Med.

[11] Thoene M, Anderson-Berry A (2021). Early enteral feeding in preterm infants: A narrative review of the nutritional, metabolic, and developmental benefits. Nutrients.

[12] Martin CR, Ling PR, Blackburn GL (2016). Review of infant feeding: Key features of breast milk and infant formula. Nutrients.

[13] (2025). The costs of inadequate breastfeeding of infants in Mexico - PubMed. https://pubmed.ncbi.nlm.nih.gov/25733643/.

[14] White H, King L (2014). Enteral feeding pumps: Efficacy, safety, and patient acceptability. Med Devices (Auckl).

[15] Klepper CM, Moore J, Gabel ME, Fleet SE, Kassel R (2023). Pediatric formulas: Categories, composition, and considerations. Nutr Clin Pract.

[16] Bakshi S, Paswan VK, Yadav SP (2023). A comprehensive review on infant formula: Nutritional and functional constituents, recent trends in processing and its impact on infants' gut microbiota. Front Nutr.

[17] (2025). Enteral formula selection: A review of selected product categories. https://www.researchgate.net/publication/285118304_Enteral_formula_selection_A_review_of_selected_product_categories.

[18] Brink LR, Lönnerdal B (2020). Milk fat globule membrane: The role of its various components in infant health and development. J Nutr Biochem.

[19] Prell C, Koletzko B (2016). Breastfeeding and complementary feeding: Recommendations on infant nutrition. Dtsch Arztebl Int.

[20] Lemaire M, Le Huërou-Luron I, Blat S (2018). Effects of infant formula composition on long-term metabolic health. J Dev Orig Health Dis.

[21] Doley J (2022). Enteral nutrition overview. Nutrients.

[22] Shrager S, Adigun A, Motolongo S, Santos CS, Rowe-King P, Duro D (2023). Comparison of home-blenderized formula and commercial enteral formulas for gastrostomy tube-fed children: A retrospective, prospective cohort study. Cureus.

[23] Alabbas F, Dumant C (2022). Outcomes of blenderized gastrostomy feeding in children at Rouen University Hospital. Pediatric Health Med Ther.

[24] Schmitz ÉPC, Silva EC, Lins Filho OL, Antunes MM, Brandt KG (2021). Blenderized tube feeding for children: An integrative review. Rev Paul Pediatr.

[25] Katagiri S, Ohsugi Y, Shiba T (2023). Homemade blenderized tube feeding improves gut microbiome communities in children with enteral nutrition. Front Microbiol.

[26] Hong SJ (2018). Types of special infant formulas marketed in Korea and their indications. Pediatr Gastroenterol Hepatol Nutr.

[27] Verduci E, Salvatore S, Bresesti I (2021). Semi-elemental and elemental formulas for enteral nutrition in infants and children with medical complexity-Thinking about cow's milk allergy and beyond. Nutrients.

[28] Mukai J, Miyanaga Y, Ishizaka T, Asaka K, Nakai Y, Tsuji E, Uchida T (2004). Quantitative taste evaluation of total enteral nutrients. Chem Pharm Bull.

[29] Jesuit C, Dillon C, Compher C, Lenders CM (2010). A.S.P.E.N. clinical guidelines: Nutrition support of hospitalized pediatric patients with obesity. JPEN J Parenter Enteral Nutr.

[30] Vandenplas Y, Brough HA, Fiocchi A (2021). Current guidelines and future strategies for the management of cow's milk allergy. J Asthma Allergy.

[31] (2025). A partially hydrolyzed whey formula provides adequate nutrition in high-risk infants for allergy - PMC. https://pmc.ncbi.nlm.nih.gov/articles/PMC9149326/..

[32] Dias JA, Santos E, Asseiceira I, Jacob S, Koninckx CR (2022). The role of infant formulas in the primary prevention of allergies in non-breastfed infants at risk of developing allergies—Recommendations from a multidisciplinary group of experts. Nutrients.

[33] Cabana MD (2017). The role of hydrolyzed formula in allergy prevention. Ann Nutr Metab.

[34] Rosen MJ, Dhawan A, Saeed SA (2015). Inflammatory bowel disease in children and adolescents. JAMA Pediatr.

[35] Scarallo L, Lionetti P (2021). Dietary management in pediatric patients with Crohn's Disease. Nutrients.

[36] Lahad A, Weiss B (2015). Current therapy of pediatric Crohn's disease. World J Gastrointest Pathophysiol.

[37] Muir A, Falk GW (2021). Eosinophilic esophagitis: A review. JAMA.

[38] Shulhan J, Dicken B, Hartling L, Larsen BM (2017). Current knowledge of necrotizing enterocolitis in preterm infants and the impact of different types of enteral nutrition products. Adv Nutr.

[39] de Lange IH, van Gorp C, Eeftinck Schattenkerk LD, van Gemert WG, Derikx JP, Wolfs TG (2021). Enteral feeding interventions in the prevention of necrotizing enterocolitis: A systematic review of experimental and clinical studies. Nutrients.

[40] Fallon EM, Nehra D, Potemkin AK, Gura KM, Simpser E, Compher C, Puder M (2012). A.S.P.E.N. clinical guidelines: Nutrition support of neonatal patients at risk for necrotizing enterocolitis. JPEN J Parenter Enteral Nutr.

[41] Kim CS, Claud EC (2019). Necrotizing enterocolitis pathophysiology: How microbiome data alter our understanding. Clin Perinatol.

[42] Puoti MG, Köglmeier J (2022). Nutritional management of intestinal failure due to short bowel syndrome in children. Nutrients.

[43] Mariotti Zani E, Grandinetti R, Cunico D, Torelli L, Fainardi V, Pisi G, Esposito S (2023). Nutritional care in children with cystic fibrosis. Nutrients.

[44] Brown B, Roehl K, Betz M (2015). Enteral nutrition formula selection: Current evidence and implications for practice. Nutr Clin Pract.

[45] Hernell O (2012). Current safety standards in infant nutrition--a European perspective. Ann Nutr Metab.

[46] Stolwijk NN, Bosch AM, Bouwhuis N (2023). Food or medicine? A European regulatory perspective on nutritional therapy products to treat inborn errors of metabolism. J Inherit Metab Dis.

